# Phylogenetic analysis of eukaryotic NEET proteins uncovers a link between a key gene duplication event and the evolution of vertebrates

**DOI:** 10.1038/srep42571

**Published:** 2017-02-16

**Authors:** Madhuri A. Inupakutika, Soham Sengupta, Rachel Nechushtai, Patricia A. Jennings, Jose’ N. Onuchic, Rajeev K. Azad, Pamela Padilla, Ron Mittler

**Affiliations:** 1 Department of Biological Sciences, University of North Texas, Denton TX 76203, USA; 2 The Alexander Silberman Institute of Life Science, Hebrew University of Jerusalem, Edmond J. Safra Campus at Givat Ram, Jerusalem 91904, Israel; 3 Department of Chemistry & Biochemistry, University of California at San Diego, La Jolla, CA 92093, USA; 4 Center for Theoretical Biological Physics and Department of Physics and Astronomy, Chemistry and Biosciences, 239 Brockman Hall, 6100 Main Street- MS-61, Rice University, Houston, TX 77005, USA; 5 Department of Mathematics, University of North Texas, Denton, TX 76203, USA

## Abstract

NEET proteins belong to a unique family of iron-sulfur proteins in which the 2Fe-2S cluster is coordinated by a CDGSH domain that is followed by the “NEET” motif. They are involved in the regulation of iron and reactive oxygen metabolism, and have been associated with the progression of diabetes, cancer, aging and neurodegenerative diseases. Despite their important biological functions, the evolution and diversification of eukaryotic NEET proteins are largely unknown. Here we used the three members of the human NEET protein family (CISD1, mitoNEET; CISD2, NAF-1 or Miner 1; and CISD3, Miner2) as our guides to conduct a phylogenetic analysis of eukaryotic NEET proteins and their evolution. Our findings identified the slime mold *Dictyostelium discoideum*’s CISD proteins as the closest to the ancient archetype of eukaryotic NEET proteins. We further identified CISD3 homologs in fungi that were previously reported not to contain any NEET proteins, and revealed that plants lack homolog(s) of CISD3. Furthermore, our study suggests that the mammalian NEET proteins, mitoNEET (CISD1) and NAF-1 (CISD2), emerged via gene duplication around the origin of vertebrates. Our findings provide new insights into the classification and expansion of the NEET protein family, as well as offer clues to the diverged functions of the human mitoNEET and NAF-1 proteins.

NEET proteins belong to a newly discovered class of iron-sulfur (Fe-S) proteins that harbor the 3Cys-1His CDGSH 2Fe-2S binding domain [**C**-X-**C**-X2-(S/T)-X3-P-X-**C**-D-G-(S/A/T)-**H**], followed by the “NEET” motif^1^. They were originally classified as zinc-finger proteins based on the presence of the *zf-*CDGSH domain (originally identified as a zinc-finger motif), but were later discovered to contain an 2Fe-2S cluster bound to the 3Cys-1His coordinates of the CDGSH motif by biochemical and X-ray structural analysis^2^,^3^,^4^,^5^,^6^. NEET proteins are unique among Fe-S proteins because their 3Cys-1His cluster coordination structure allows them to be both relatively stable, as well as to easily donate their 2Fe-2S cluster to other cluster acceptor proteins^7^,^8^,^9^. This feature makes NEET proteins highly versatile in their biological functions and has led to the idea that they participate in the regulation of iron, Fe-S, reactive oxygen species (ROS), and/or redox metabolism of cells^1^,^10^,^11^,^12^,^13^,^14^,^15^,^16^,^17^,^18^,^19^,^20^,^21^,^22^,^23^. Gain- and loss-of-function analysis of mammalian and plant NEET proteins indeed revealed that the plant and mammalian proteins have at least one conserved function in maintaining the overall iron and ROS homeostasis of cells, and in particular that of the mitochondria^1^,^19^,^21^,^24^.

NEET proteins can be classified into two types: Class I NEET proteins containing a single copy of the CDGSH 2Fe-2S binding motif per polypeptide chain, and Class II NEET proteins containing two copies of the CDGSH motif within a single polypeptide chain. Class I NEET proteins function in cells as a homodimer that is anchored to a membrane, and Class II NEET proteins function as a soluble monomer^1^,^25^. In humans, Class I NEET proteins are encoded by two genes: CISD1 that encodes mitoNEET (mNT), a protein that is anchored to the mitochondrial outer membrane, and CISD2 that encodes NAF-1 (previously called Miner1), a protein that is anchored to the ER, mitochondria and their interacting membranes (MAM)^1^. The Class II NEET protein in humans is encoded by the CISD3 (also called Miner 2) gene, and its protein product, which is localized to the mitochondria, does not contain a membrane anchoring domain^1^,^25^. Human mNT (CISD1) and NAF-1 (CISD2) proteins share 54% identical residues and 69% similar residues (sharing similar physicochemical properties) over 99% of their sequence lengths (Supplementary Fig. S1). In contrast, human CISD3 shares 50% identical and 63% similar residues with mNT over 50% of its sequence length, and 38% identical and 50% similar residues with NAF-1 over 63% of its sequence length (Supplementary Fig. S1).

Recent studies revealed that NEET proteins play important roles in several different human diseases. For example, mNT was implicated in diabetes, obesity, and cancer^1^,^16^,^17^, and NAF-1 was implicated in BCL-2-Beclin-1-BIK-dependent autophagy and BCL-2-dependent apoptosis, as well as in neurodegenerative diseases, skeletal muscle maintenance, cancer, and aging^1^,^10^,^11^,^12^,^13^,^14^,^15^,^17^,^18^,^21^,^22^,^23^. In addition, a homozygous intragenic deletion, or a missense mutation, that abolishes NAF-1 function leads to a rare genetic disease called Wolfram Syndrome 2 (WFS2); phenotypes associated with this disease include hearing deficiencies, neurodegeneration, severe blindness, diabetes and a lower life expectancy^1^,^23^.

Due to the importance of NEET proteins to human health^1^,^10^,^11^,^12^,^13^,^14^,^15^,^17^,^18^,^21^,^22^,^23^, their cluster transfer flexibility potential that was found to be critical for their function in cancer cells^24^, and their apparent presence in different unicellular and multicellular organisms^1^,^2^,^3^,^4^,^5^,^7^,^8^,^9^,^10^,^11^,^12^,^13^,^14^,^15^,^16^,^17^,^18^,^19^,^20^,^21^,^22^,^23^,^25^, we decided to conduct a phylogenetic analysis of NEET proteins in eukaryotic organisms. In particular, we were interested to find how many different NEET variants exist in different species, what is the origin of the human CISD genes, and why NEET proteins are absent in fungi, as reported in the previous studies^25^. Providing an answer to these questions would help in choosing different model organisms to study NEET protein function, as well as shed light on the different roles the different human NEET proteins play in cells.

## Results

**Figure 1:**
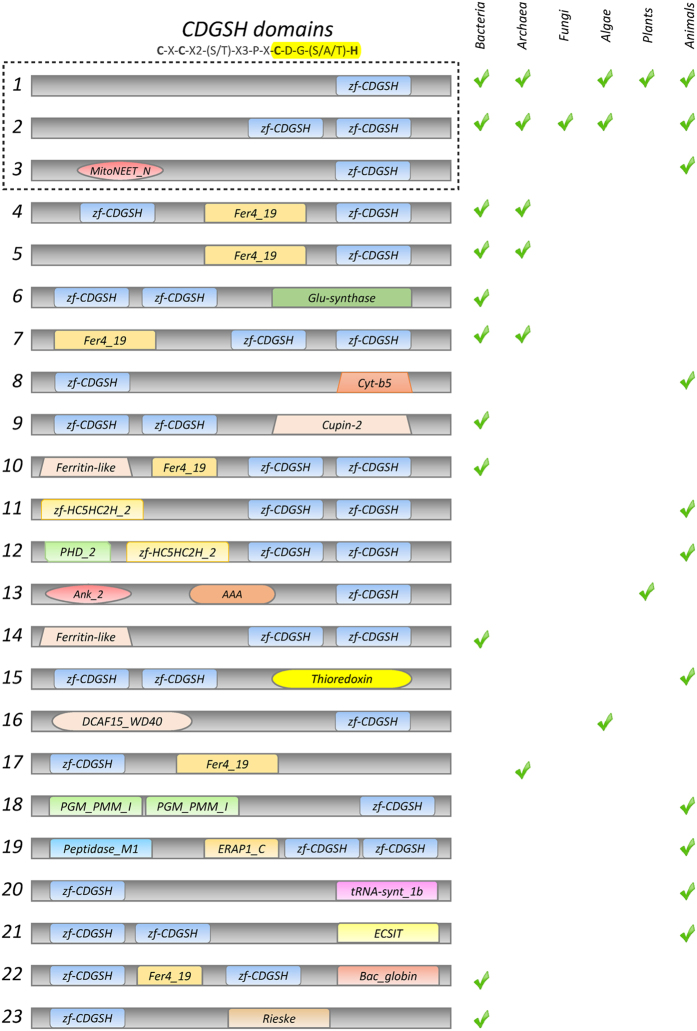
z*f*-CDGSH domain organization and architecture across different lineages. The conserved sequence C-X-C-X2-(S/T)-X3-P-X-C-D-G-(S/A/T)-H is a defining feature of the CDGSH protein family (3Cis-1His coordinates are in bold and the CDGSH motif is highlighted in yellow). The presence or absence of each protein type in bacteria, archaea, fungi, algae, plants and animals is indicated on the right. Human CISD1/CISD2 NEET proteins belong to groups 1 and 3, and human CISD3 NEET protein belongs to group 2. The three human CDGSH NEET proteins (represented by groups 1–3; dashed box) were used for all BLAST searches and phylogenetic tree analysis of NEET proteins in this work.

To conduct a phylogenetic analysis of CDGSH-motif containing NEET proteins, we first examined how many different types of proteins contain the *zf-*CDGSH motif. At least 23 different proteins containing the CDGSH motif were found in the Pfam^26^ database (Fig. 1). These varied from a single or a double motif of CDGSH to combinations of the CDGSH motif(s) with other domains such as thioredoxin, ferritin-like, and Glu-synthase. Although many different proteins were found to contain multiple copies of a particular domain, no protein variants with three or more copies of the CDGSH motif were found by our search. The reason for this is currently unknown, but it could be related to either the function of this motif as a putative Fe-S binding domain involved in cluster transfer reactions, or the ability of this motif to oligimerize^1^. The large number of different proteins containing the CDGSH motif, coupled with the uncertainty of how many of these proteins are Fe-S proteins as opposed to zinc-finger proteins, prompted us to conduct our phylogenetic analysis using the three well-defined human CDGSH-NEET proteins, that were biochemically and structurally shown to harbor an Fe-S cluster^1^, as guides (Fig. 1). This strategy provided us with a set of homologs of the three human NEET proteins in different species. The three human NEET proteins are represented in Fig. 1 as CDGSH variants 1 and 3 (CISD1 and CISD2), and 2 (CISD3), respectively (highlighted with a dashed box in Fig. 1). It should be noted that the domain annotated as MitoNEET_N in the Pfam database appears in both the human mNT (CISD1) and the NAF-1 (CISD2) proteins.

To identify homologs of human CISD1-, CISD2- and CISD3-NEET proteins in different organisms from different lineages, we first determined the thresholds for the PSI-BLAST searches to be used in our analysis. For this purpose we conducted a sensitivity analysis. Human CISD1, CISD2 and CISD 3 sequences were compared to each other using different PSI-BLAST parameters and the PSI-BLAST parameters at which any of these sequences, when used as the query sequence, returned the other two sequences among the BLAST hits were determined (Supplementary Table S1). Based on this analysis we used an Expect threshold (e-value) of 10 and a PSI-BLAST threshold of 5 for our PSI-BLAST^27^ searches for the three human NEET proteins (CISD1–3) in each of the genomes analyzed.

**Figure 2:**
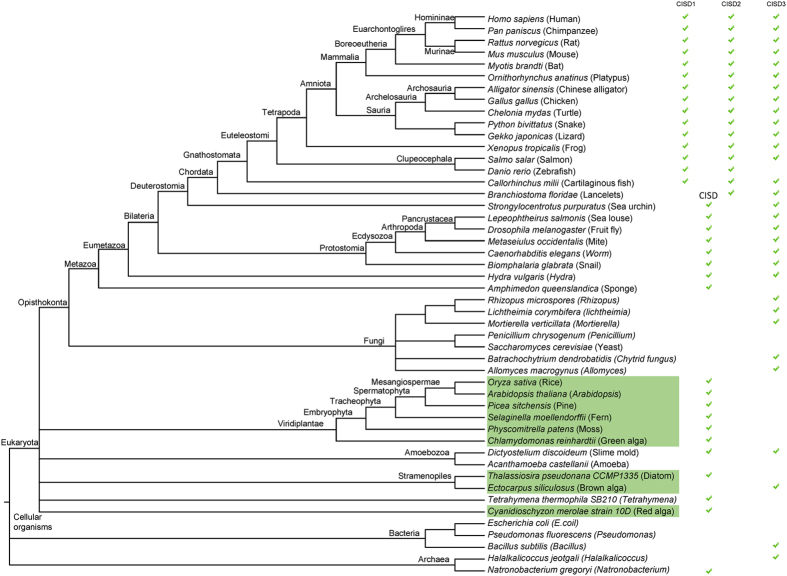
Occurrence of CISD, CISD1, CISD2 and CISD3 proteins in different species. A taxonomy common tree of species was obtained from NCBI (NCBI Taxonomy). The tree was populated with one representative fully sequenced genome on each of its branches (Supplementary Table S2). Each genome was then subjected to a PSI-BLAST search with each of the different human CISD sequences and the presence or absence of human CISD homologs is indicated on right. When a clear distinction could not be made between homologs of CISD1 or CISD2 with a 50% similarity cutoff to the two different proteins, the homolog was annotated as CISD. All CISD, CISD1, and CISD2 homologs contain a single copy of the CDGSH domain per polypeptide chain, and all homologs of CISD3 contain two. Oxygen-evolving photosynthetic organisms are highlighted with a green background.

To examine the phyletic pattern of NEET proteins, that is, the presence or absence of NEET protein variants in organisms from different lineages, we retrieved a common tree of species from the NCBI taxonomy site (http://www.ncbi.nlm.nih.gov/Taxonomy/CommonTree/wwwcmt.cgi), and populated it with organisms with fully sequenced and annotated genomes (retrieved from: http://www.ncbi.nlm.nih.gov/genome/browse/; Supplementary Table S2). For each branch on the tree we included only a single representative organism with a fully sequenced genome (Fig. 2). We focused on eukaryotes, with prokaryotes represented by a few bacterial and archaeal species. Each genome represented on the species tree was individually subjected to a protein PSI-BLAST search using human CISD1, CISD2, or CISD3 as a query, and the presence or absence of each of the different CISD homologs was determined and indicated next to the organism name on the species tree (Fig. 2). If a NEET protein homolog could not be unambiguously classified as CISD1 or CISD2 (i.e., its similarity was below a 50% cut-off to each of these proteins), it was annotated as CISD (Fig. 2). As shown in Fig. 2, we could only clearly distinguish between CISD1 and CISD2 clades in vertebrates (Chordata), suggesting that the gene duplication that resulted in the emergence of CISD1 and CISD2 likely coincided with the origin of vertebrates. Interestingly, we could identify several fungi that contained homologs of CISD3 (see also Fig. 1). However, we did not find CISD3 homologs in plants and this was further verified by performing a PSI-BLAST search for human CISD3 in all plant sequences (Supplementary Fig. S2; the only hits we got were of one of the CDGSH domains of human CISD3 with the single CDGSH domain found in the plant Class I CISD proteins). Although most organisms represented on the species tree shown in Fig. 2 contain at least one homolog of NEET proteins, some organisms, for example, *Saccharomyces cerevisiae* (fungi), or *Acanthamoeba castellanii* (amoeba) do not appear to have homologs of NEET proteins. Likewise, some bacteria such as *Escherichia coli* or *Pseudomonas fluorescens* do not harbor homologs of NEET proteins. These findings suggest that although NEET proteins are highly conserved in most multicellular organisms, they may not be essential for some eukaryotes or prokaryotes.

**Figure 3:**
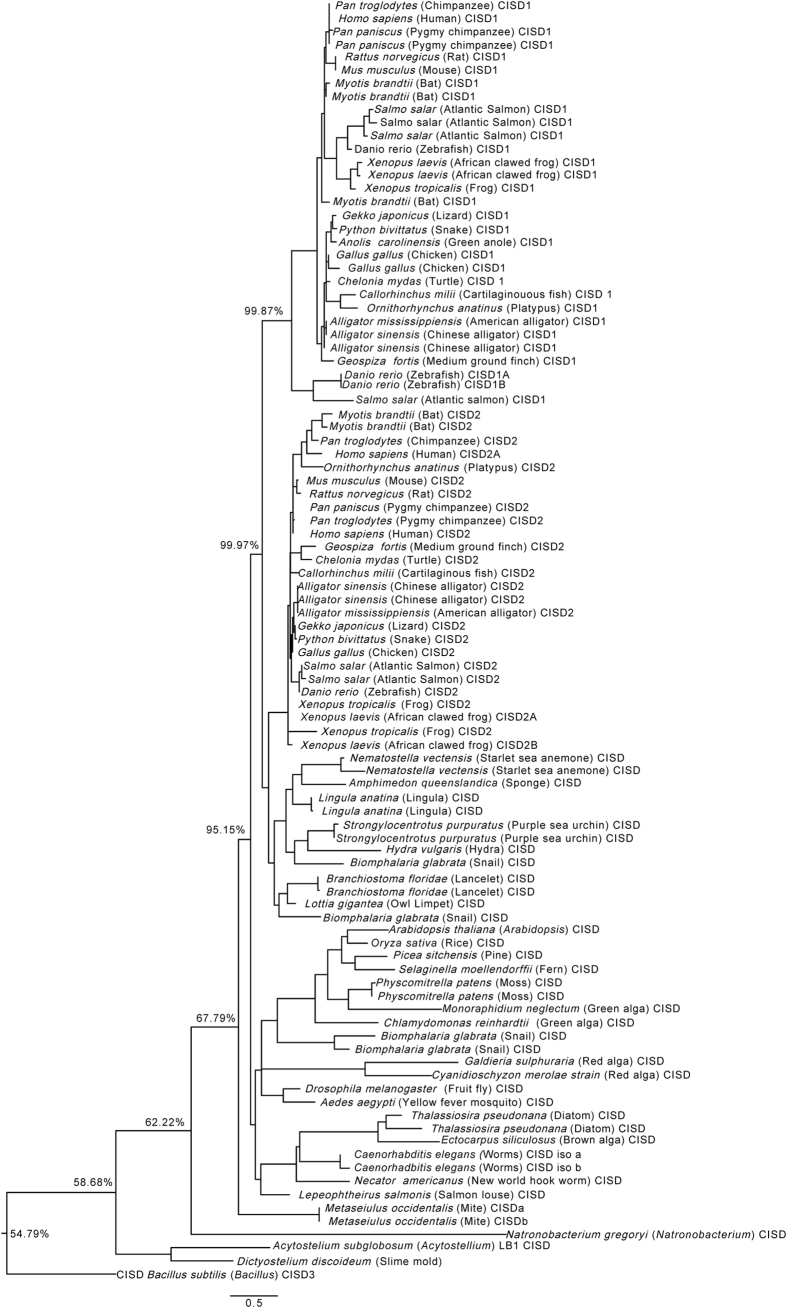
Phylogenetic tree constructed for all CISD, CISD1 and CISD2 proteins, with a single copy of the CDGSH domain per polypeptide chain, found in two different representative fully sequenced genomes for each of the branches of the taxonomy common tree of species presented in Fig. 2 (Supplementary Tables S2 and S3). All protein sequences were obtained from the NCBI protein database using the PSI-BLAST algorithm with PSI threshold value 5 and e-value 10. The sequences were then aligned using the software MUSCLE. trimAL was employed to eliminate poorly aligned regions in the alignment. A maximum-likelihood phylogenetic tree with posterior probability support was then created using the PhyML program. The tree was finally edited with the software FigTree 1.4.0. Multiple sequence alignments and a version of the tree with complete protein annotations and posterior probabilities are included in Supplementary Figs S3 and S5, respectively.

**Figure 4:**
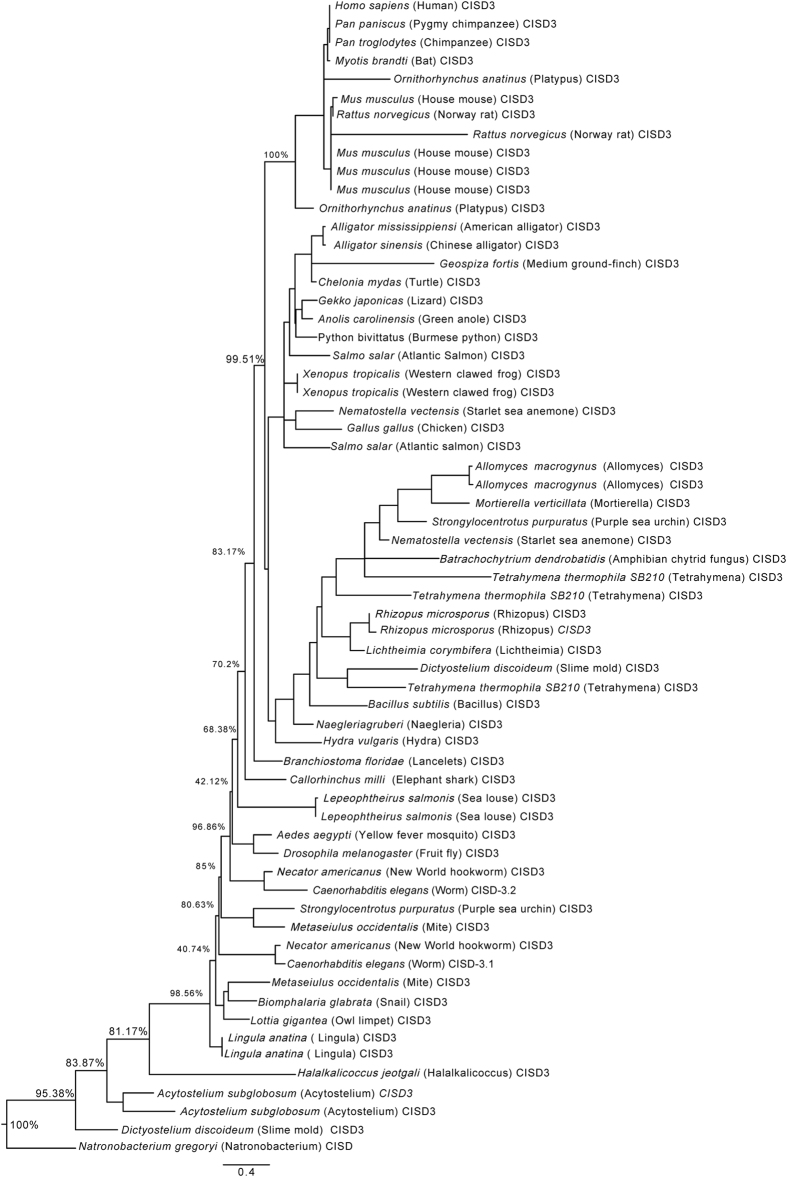
Phylogenetic tree constructed for all CISD3 proteins with two copies of the CDGSH domain per polypeptide chain found in two different representative fully sequenced genomes for each of the branches of the taxonomy common tree of species presented in Fig. 2 (Supplementary Tables S2 and S3). All protein sequences were obtained from the NCBI protein database using the PSI-BLAST algorithm with PSI threshold value 5 and e-value 10. The sequences were then aligned using the software MUSCLE and trimAL was used to delete regions with too many gaps. A maximum-likelihood phylogenetic tree with posterior probability support was then created using the PhyML program. The tree was finally edited with the software FigTree 1.4.0. Multiple sequence alignments and a version of the tree with complete protein annotations and posterior probabilities are included in Supplementary Figs S4 and S6, respectively.

To further uncover the evolution the NEET protein family in eukaryotes, we performed multiple sequence alignments (MSAs; Supplementary Figs S3 and S4) and constructed phylogenetic trees for CISD1 and CISD2 homologs (Class I; Fig. 3 and Supplementary Fig. S5), and CISD3 homologs (Class II; Fig. 4 and Supplementary Fig. S6) using a maximum likelihood method (detailed in the Methods section). For the phylogenetic analyses of protein sequences for organisms shown in Figs 3, 4, and Supplementary Figs S3–S6, and to increase the sensitivity of these trees, we used two different organisms with a fully sequenced genome for each branch of the species tree (Fig. 2) and included all protein sequences that were identified from these genomes with the CISD1/CISD2/CISD3 PSI-BLAST search described above (these sometimes included different protein sequences that originated from the same gene via alternative splicing; Supplementary Table S3). As shown in Fig. 3, CISD1 and CISD2 protein sequences formed two distinct clades, highlighting within and between clade relationships among the CISD1/CISD2 sequences, and distinguishing the CISD1 and CISD2 sequences of vertebrates from the rest of the CISD sequences (Fig. 2). Interestingly, the plant, insect and worm CISDs were closer to the CISD2 clade than to the CISD1 clade, whereas the slime molds CISDs were very distinct from either of the CISD1 or CISD2 clades, with more proximity to that of the outlier (*Bacillus* CISD3). These findings suggest that the slime mold CISD protein could represent an early or ancient version of eukaryotic CISDs before the emergence of CISD1 and CISD2 genes. A similar analysis performed for all protein sequences with homology to human CISD3 (all containing two copies of the CDGSH domain within a single protomer) revealed that, with the exception of lancelets and elephant shark, all vertebrate CISD3s grouped as a distinct clade. Interestingly, the CISD3 proteins of some organisms that contained more than one CISD3 protein in their genome (e.g., slime mold and worm) did not group together, suggesting that the two different CISD3 proteins found in these organisms might have acquired different functions during evolution (Fig. 4). In general, compared to CISD1 and 2 proteins (Fig. 3), CISD3 proteins from different organisms (Fig. 4) displayed a high degree of divergence in structure and function. As with CISD1 and 2 (Fig. 3), at least one of the slime mold CISD3 proteins was very distinct from the rest of the CISD3 clades, with more proximity to the outlier (Archaea CISD; Fig. 4).

**Figure 5:**
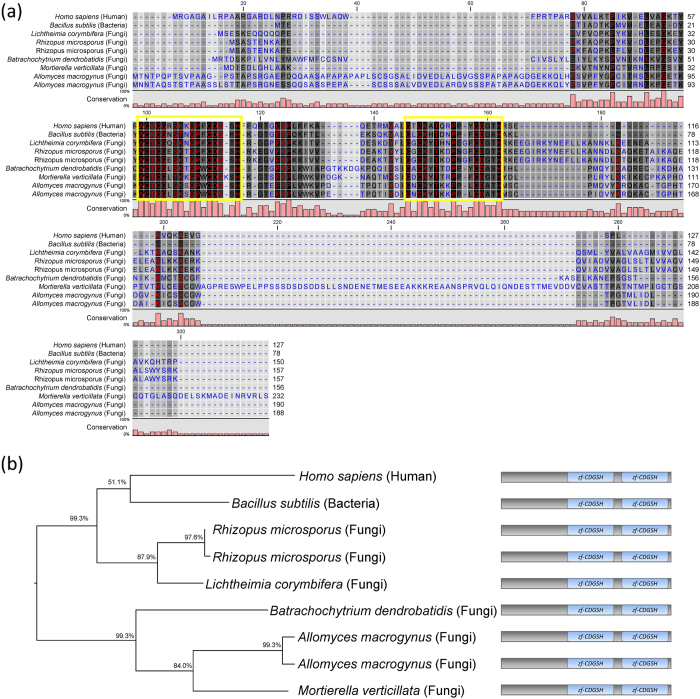
Analysis of fungi CISD genes. (**a**) Multiple sequence alignment of the CISD3 proteins from different fungi, bacteria and human generated using MUSCLE. Yellow boxes indicate the CDGSH domains. Bar graph under the aligned sequences indicates degree of conservation (%). Color legend: *Background*: White - Least conserved, Black - Most conserved; *Font*: Blue - Least conserved; Red - Most conserved. (**b**) Maximum-likelihood tree of CISD3 proteins of fungi, bacteria and human generated using PhyML. All the sequence have the same domain architecture as represented in the figure.

Surprisingly, and contrary to previous reports^25^, our study revealed the presence of CISD3 proteins in fungi. To confirm that the fungal CISD3 sequences identified were indeed CISD-like proteins we aligned all fungal CISD proteins with the CISD3 proteins of human and bacteria (*Bacillus subtilis*). As shown in Fig. 5a, all fungal CISD3-like sequences contained two highly conserved CDGSH domains confirming that they are indeed CISD3 homologs. A phylogenetic tree constructed for the fungal, human, and bacterial CISD3 proteins further revealed that fungal CISD3-like genes belonged to two distinct groups- one that shares similarity with human and bacteria, and the other that is more distinct (Fig. 5b). These findings support the existence of NEET proteins in parasitic as well as free-living fungi and suggest that fungal NEET proteins could have diverged in their functions to facilitate adaptation to their hosts or environments.

## Discussion

The conserved structure of the CDGSH domain allowed us to conduct a comparative phylogenetic analysis of NEET proteins primarily focusing on eukaryotic organisms. A previous analysis of CDGSH proteins in prokaryotes identified many different types of CDGSH proteins with a single or double CDGSH domain, but did not address the complex nature of CISD-like proteins in eukaryotic organisms^25^. A key finding of our analysis was the identification of the vertebrate origin as the putative point of gene duplication that yielded the two different mammalian CISD proteins: CISD1 (mNT) and CISD2 (NAF-1) (Fig. 2). The emergence of vertebrates was accompanied by several important cellular and developmental milestones. These included among others the origin of an adaptive immune system, the emergence of neural crest cells and complex neuronal networks, and the appearance of synchronized and complex symmetric segmentation patterns^28^,^29^,^30^. Because NAF-1 is linked to several different neurological disorders^1^,^10^,^11^,^13^,^18^, it is plausible that the origin of NAF-1 through ancestral CISD duplication and differentiation coincided with the origin of specialized neuronal cells in vertebrates, and that NAF-1 conferred important adaptive functions for the maintenance of these neuronal cells. This could be reflected by the important role NAF-1 currently plays in neurodegenerative diseases. Another possibility, which could be associated with NAF-1 role in the regulation of apoptosis and autophagy^1^,^11^,^12^,^15^,^21^, might be evolutionary linked to the appearance of the adaptive immune system and the utilization of cell death pathways by lymphocytes, as part of this system. Further studies are of course needed to establish the evolutionary significance of mNT and NAF-1 functions in vertebrates. With respect to the putative duplication event that resulted in CISD1 and CISD2, it is worth noting that although the CISD phyletic pattern overlaid on the species tree indicates that this duplication event is likely linked to the emergence of vertebrates (Fig. 2), the phylogenetic tree analysis of CISD1 and CISD2 from different organisms (Fig. 3) showed one clade of CISD proteins from snail, lancelet, hydra, lingual, sponge and sea anemone to be more closely related to CISD2 than to CISD1. This finding could suggest that CISD2 evolved first, before the vertebrates emerged, and that CISD1 appeared via gene duplication around or after the radiation of vertebrates. Of course this observation could also reflect a discrepancy between the phylogenetic gene tree and the species tree, arising as a consequence of factors such as incomplete lineage sorting, recombination, horizontal gene transfer, etc, or the inability of the currently available data to resolve the gene tree^31^,^32^.

In addition to inferring the divergence of mNT and NAF-1 using our species and gene tree analysis, our study also revealed the presence of CISD3 genes in fungi. Fungal CISD3 proteins display high sequence similarity to human CISD3 and are present in at least 5 species of fungi (Fig. 5). Because fungi lack CISD1- or CISD2-like proteins, and some do not appear to have any type of CISD protein, it is possible that only certain types of fungi with specialized requirements retained the CISD3 gene, while others lost it completely. It would be of interest in future studies to decipher the common features and growth requirements that distinguish the fungi that contain CISD proteins from the ones that do not. The possibility that some organisms lost a specific class of CISD proteins is further highlighted by our striking finding that plants do not contain the homologs of CISD3 (Fig. 2). Because plants contain chloroplasts, that took over during evolution some of the biosynthetic and metabolic pathways that are common to mitochondria in animals, and because the plant CISD protein (e.g., AtNEET^19^) is associated with both chloroplasts and mitochondria, it is possible that CISD3 function in mitochondria (that is largely unknown at present) is not required in plants.

Perhaps one of the most interesting questions, when dealing with a phylogenetic analysis of a protein family, is - what is the most ancestral form of the family? From the phylogenetic standpoint of eukaryotic CISD evolution it appears from our analysis that the slime mold *Dictyostelium discoideum*’s CISD proteins (with a single or double copy of the CDGSH domain per protomer) are the closest to the archetype of eukaryotic Class I and Class II CISD proteins. These proteins were found to be closest to the outliers in our phylogenetic analysis of CISD1/2 and CISD3 proteins (Figs 3 and 4, respectively). Of course from the standpoint of evolution of prokaryotic and eukaryotic organisms it is much harder to determine what is the most ancestral form of all CISDs, aside from speculating that the proto-CISD had only one copy of the CDGSH domain and that it either evolved to form a single-copy CDGSH CISD-like protein, or underwent a CDGSH domain duplication to yield a CISD3-like protein. Another possibility is of course that a CISD3-like ancestral protein (containing two CDGSH domains) was duplicated, with each gene copy losing one of its CDGSH domains to form CISD1- and CISD2- single CDGSH domain-like proteins that would enable a higher degree of cooperativity and regulation in their interaction and function, similar to the mammalian NAF-1 and mNT^1^. In this context it is worthwhile to note that both bacteria and archaea were found to contain members of Class I (single CDGSH domain) or Class II (two CDGSH domains) of the CISD family of proteins (Figs 1 and 2)^25^. Because CISD1 (mNT) and CISD3 localize to mitochondria in eukaryotes, whereas CISD2 (NAF-1) is primarily localized to the ER and was shown to have more diverged functions than CISD1 or CISD3^ ^1,11,12,13,15,17,22,23, it is also tempting to speculate that the duplication of the Class I CISD gene resulting in the emergence of CISD1 and CISD2 proteins was followed by the acquisition of additional roles and functions by CISD2 (NAF-1) that coincided with the evolution of vertebrates as described above. The path of CISD evolution was therefore paved by important gene duplication events (i.e., the appearance of mNT and NAF-1), as well as gene deletions and loss of function (e.g., the absence of CISD3 in plants and some fungi). Further studies are of course required to identify the molecular, biochemical and environmental factors that affected the evolution of CISD proteins.

## Methods

### Selection of organisms for analysis

To determine the presence or absence of different NEET proteins in organisms from different lineages, we first retrieved a common tree of species from the NCBI taxonomy site (http://www.ncbi.nlm.nih.gov/Taxonomy/CommonTree/wwwcmt.cgi), and then selected representative organisms with fully sequenced and annotated genomes (obtained from: http://www.ncbi.nlm.nih.gov/genome/browse/; Supplementary Table S2). A total of 43 eukaryotes, 3 bacteria and 2 archaea were represented as shown in Fig. 2. As indicated in Supplementary Table S2, some of the genomes used were assembled based on reference genomes and some were assembled *de novo*.

### Protein sequence retrieval

The complete protein sequences of human CISD1, CISD2, and CISD3 were retrieved from the NCBI database (http://www.ncbi.nlm.nih.gov/protein). We used these as queries in a PSI-BLAST^27^ search to obtain CISD homologs from the genomes of the organisms selected for our analysis (Supplementary Table S2). To determine the thresholds for the PSI-BLAST searches we conducted a sensitivity analysis. Thus, CISD1, CISD2 and CISD 3 were compared to each other using different PSI-BLAST parameters to determine the parameter setting where the CISD1, CISD2, and CISD3 are retrieved as mutual blast hits of each other (Supplementary Table S1). Based on this analysis we used an Expect threshold (e-value) of 10 and a PSI-BLAST threshold of 5 for our PSI-BLAST^27^ searches for the three human NEET proteins (CISD1-3) in each of the genomes analyzed. We further generated a maximum likelihood tree using human CISD1, CISD2 and CISD3 protein sequences (Supplementary Fig. S1). This tree showed a high similarity between CISD1 and CISD2 and a low similarity between CISD1 or CISD2 and CISD3, which necessitated the more relaxed PSI-BLAST thresholds for our analysis. The PSI-BLAST parameters determined from our sensitivity analysis were therefore set to ensure that the program returns as “hits” all three human CISD sequences when any of the human CISD sequences is used as a query sequence. Using human CISD1, CISD2, and CISD3 as queries, PSI-BLAST searches were conducted against the non-redundant (NR) database of completely sequenced genomes (http://www.ncbi.nlm.nih.gov/genome/browse/; Supplementary Table S2). Iterative PSI-BLAST searches were further performed until no new CISD homologs were found. The candidate CISD 1, CISD2, CISD3 sequences obtained were further examined for the presence of the signature *zf*-CDGSH domain, of which the conserved sequence **C**-X-**C**-X2-(S/T)-X3-P-X-**C**-D-G-(S/A/T)-**H** is a defining feature^1^. We utilized the services of PFAM^26^ and InterProScan^33^ for this analysis. Partial sequences and those lacking the CDGSH domain were eliminated manually. This procedure yielded a dataset of 96 CISD1 and CISD2 candidates and 60 CISD3 candidates that were used for our phylogenetic study.

### PFAM domain architectures

The unique domain architectures of *zf*-CDGSH were obtained from the PFAM database. A total of 489 sequences across 274 species containing one of the 23 domain architectures were recorded in the PFAM database, as shown in Fig. 1.

### Sequence alignment and phylogenetic analysis

Multiple sequence alignments of the 96 candidate CISD1 and CISD2 proteins, and of the 60 candidate CISD3 proteins, or all of the candidate CISD proteins, were performed by command-line MUSCLE^34^ with default options. For trimming poorly aligned regions, trimAL was employed (-automated1 option) to generate better quality alignments^35^.

PhyML version 3.0 was employed to construct phylogenetic trees using a maximum-likelihood method^36^. Trees were built for CISD1 and CISD2 protein sequences, and for CISD3 sequences. For statistical reliability, the following test or parameters were used: posterior probability distribution on trees, and an approximate likelihood-ratio test (aLRT) based on logarithm of the ratio of likelihood computed for the current tree and that of the best alternative. To estimate the optimal model of substitution, ProtTest was used for each alignment^37^. ProtTest indicated the VT amino acid model with gamma distribution shape parameter (VT + G) as the best fitting model among the 112 examined evolutionary models, based on Akaike information criterion (AIC) statistics. The maximum likelihood trees were generated using the VT + G model. Trees were visualized and edited using the program FigTree 1.4.0 38.

## Additional Information

**How to cite this article:** Inupakutika, M. A. *et al*. Phylogenetic analysis of eukaryotic NEET proteins uncovers a link between a key gene duplication event and the evolution of vertebrates. *Sci. Rep.* **7**, 42571; doi: 10.1038/srep42571 (2017).

**Publisher's note:** Springer Nature remains neutral with regard to jurisdictional claims in published maps and institutional affiliations.

## Supplementary Material

Supplementary Figures and Tables
