# Supplementary material for: Phylogenetic analysis of eukaryotic NEET proteins uncovers a link between a key gene duplication event and the evolution of vertebrates
Source: Sci Rep. 2017 Feb 16;7:42571. doi: 10.1038/srep42571 (PMC5311916; doi:10.1038/srep42571)
Supplement: Supplementary Figures and Tables [file srep42571-s1.pdf]

Supplementary Material for:

Phylogenetic analysis of eukaryotic NEET proteins uncovers a link between a key gene duplication event and the evolution of vertebrates

Madhuri A. Inupakutika[1,a], Soham Sengupta[1,a], Rachel Nechushtai[2], Patricia A. Jennings[3], Jose' N. Onuchic[4], Rajeev K. Azad[1,5], Pamela Padilla[1] and Ron Mittler[1,*]

Supplementary Table S1. Sensitivity analysis used to set the optimum PSI-BLAST parameters. The homology between human CISD1, CISD2 and CISD3 proteins was determined (1 donates a hit, 0 donates a no hit) using a PSI-BLAST between the different proteins. Expect threshold value was set to 10 and PSI-BLAST threshold was altered until a BLAST with all three proteins identified a hits (1) with all three different proteins (i.e., all BLASTs resulted in a hit). This analysis set the lowest parameters that would obtain a hit with CISD1 or CISD2 on the more remotely homologous protein CISD3.

Supplementary Table S2. List of fully sequenced genomes used for the analysis presented in Fig. 2-5. Fully sequenced genomes were obtained from: http://www.ncbi.nlm.nih.gov/genome/browse/.

Supplementary Table S3. List of CISD homologs and their Gene IDs. Highlighted rows are product of alternative splicing. For *C. elegans* gene coordinates were obtained from wormbase (http://www.wormbase.org/). Human CISD2A was obtained from InterProScan.

Supplementary Fig. S1. Phylogenetic tree, multiple sequence alignment and percentage identity/similarity between human CISD1, CISD2 and CISD3.

Supplementary Fig. S2. NCBI PSI-BLAST result of Human CISD3 vs all higher plants, non-redundant [PSI-BLAST threshold value 5 and Expect threshold 10]. The only hit to a plant CISD protein was to the CDGSH domain of a plant protein that has only one CDGSH motif and was therefore a Class I and not a Class II homolog CISD protein that contains two CDGSH domains (like CISD3).

Supplementary Fig. S3. Multiple sequence alignment of CISD1 and CISD2 homologs generated using MUSCLE with default parameters. The bar graph represents the degree of conservation along the CDGSH domain. Color legend: *Background*: White - Least conserved, Black - Most conserved; *Font*: Blue - Least conserved; Red - Most conserved.

Supplementary Fig. S4. Multiple sequence alignment of CISD3 homologs generated using MUSCLE with default parameters. The bar graph represents the degree of conservation along the CDGSH domain. Color legend: *Background*: White - Least conserved, Black - Most conserved; *Font*: Blue - Least conserved; Red - Most conserved.

Supplementary Fig. S5. Phylogenetic tree of CISD1 and CISD2 homologs with complete protein annotations and Bayesian posterior probabilities.

Supplementary Fig. S6. Phylogenetic tree of CISD3 homologs with complete protein annotations and Bayesian posterior probabilities.

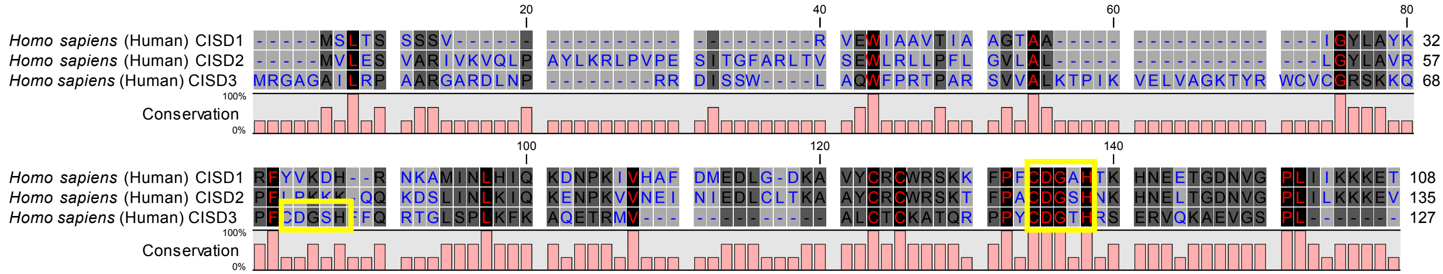

Protein	Compared to	Query Coverage (%)	Identity (%)	Similarity (%)
CISD1	CISD2	99	54	69
	CISD3	35	50	63
CISD2	CISD1	81	54	69
	CISD3	51	38	38
CISD3	CISD1	50	50	63
	CISD2	63	38	50

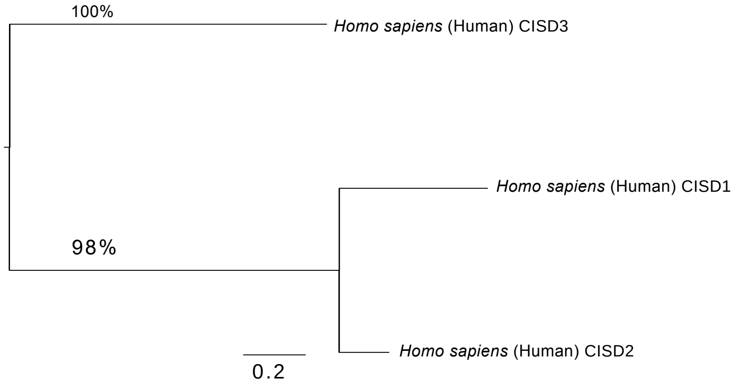

Supplementary Fig. S1. Phylogenetic tree, multiple sequence alignment and percentage identity/similarity between human CISD1, CISD2 and CISD3.

Supplementary Fig. S2. NCBI PSI‐BLAST result of Human CISD3 vs all higher plants, non-redundant [PSI‐BLAST threshold value 5 and Expect threshold 10]. The only hit to a plant CISD protein was to the CDGSH domain of a plant protein that has only one CDGSH motif and was therefore a Class I and not a Class II homolog CISD protein that contains two CDGSH domains (like CISD3).

```
CISD3 with Higher plants BLAST

Database: All non-redundant GenBank CDS
translations+PDB+SwissProt+PIR+PRF excluding environmental samples
from WGS projects
           103,788,118 sequences; 38,088,519,330 total letters
Results of PSI-Blast iteration 1
Query= CISD3

Length=127


E-value BETTER than threshold                                        Score    E
Sequences producing significant alignments:                         (Bits)  Value

XP_002457570.1  hypothetical protein SORBIDRAFT_03g009620 [Sor...  35.0    1.0
KXG32183.1  hypothetical protein SORBI_003G114400 [Sorghum bic...  35.0    1.0
XP_012852308.1  PREDICTED: CDGSH iron-sulfur domain-containing...  32.3    3.6
Sequences with E-value WORSE than threshold
XP_015060296.1  PREDICTED: uncharacterized protein LOC10700619...  32.0    5.6
XP_015940908.1  PREDICTED: uncharacterized protein LOC10746644...  32.0    9.8

ALIGNMENTS
>XP_002457570.1 hypothetical protein SORBIDRAFT_03g009620 [Sorghum bicolor]
Length=833

 Score = 35.0 bits (79),  Expect = 1.0, Method: Composition-based stats.
 Identities = 19/63 (30%), Positives = 32/63 (51%), Gaps = 6/63 (10%)

Query  17   DLNPRRDISSWLAQWFPRTPARSVVALKTPIKVELVAGKTYRWCVCGRSKKQPFCDGSHF  76
            DL+P+ D+S   A W P +  R V+ +   ++ + VA  T+       S+KQP+ D
Sbjct  53   DLSPQADVSPGRASWPPASVDRLVIIVLDALRFDFVAPSTF------FSEKQPWMDKLQV  106

Query  77   FQR  79
             Q+
Sbjct  107  LQK  109


>KXG32183.1 hypothetical protein SORBI_003G114400 [Sorghum bicolor]
Length=948

 Score = 35.0 bits (79),  Expect = 1.0, Method: Composition-based stats.
 Identities = 19/63 (30%), Positives = 32/63 (51%), Gaps = 6/63 (10%)

Query  17   DLNPRRDISSWLAQWFPRTPARSVVALKTPIKVELVAGKTYRWCVCGRSKKQPFCDGSHF  76
            DL+P+ D+S   A W P +  R V+ +   ++ + VA  T+       S+KQP+ D
Sbjct  53   DLSPQADVSPGRASWPPASVDRLVIIVLDALRFDFVAPSTF------FSEKQPWMDKLQV  106

Query  77   FQR  79
             Q+
Sbjct  107  LQK  109


>XP_012852308.1 PREDICTED: CDGSH iron-sulfur domain-containing protein NEET
[Erythranthe
guttata]
 EYU25019.1 hypothetical protein MIMGU_mgv1a016902mg [Erythranthe guttata]
Length=102
```

CISD3 with Higher plants BLAST

```
 Score = 32.3 bits (72),  Expect = 3.6, Method: Compositional matrix adjust.
 Identities = 26/87 (30%), Positives = 36/87 (41%), Gaps = 5/87 (6%)

Query  8    LRPAARGARDLNPRRDISSWLAQWFPRTPARSVVALKTPIKVELVAGKTYRWCVCGRSKK  67
            +RPA+ G     PRR ++       P         + + +  EL A     +C C RS
Sbjct  17   IRPASSGGAAAKPRRMVAVRAEAINPDIRKTEDKVVDSVVVTEL-AKPLTAYCRCWRSGT  75

Query  68   QPFCDGSHFFQR--TG--LSPLKFKAQ  90
             P CDGSH      TG  + PL  K +
Sbjct  76   FPLCDGSHVKHNKATGDNIGPLLLKNK  102


>XP_015060296.1 PREDICTED: uncharacterized protein LOC107006195 [Solanum pennellii]
Length=133

 Score = 32.0 bits (71),  Expect = 5.6, Method: Compositional matrix adjust.
 Identities = 13/30 (43%), Positives = 19/30 (63%), Gaps = 0/30 (0%)

Query  88   KAQETRMVALCTCKATQRPPYCDGTHRSER  117
            + Q  +++    CKA +R  +CDG HRSER
Sbjct  64   RQQMQQIIKDNLCKAQERMKHCDGLHRSER  93


>XP_015940908.1 PREDICTED: uncharacterized protein LOC107466447 isoform X1 [Arachis
duranensis]
Length=866

 Score = 32.0 bits (71),  Expect = 9.8, Method: Composition-based stats.
 Identities = 17/46 (37%), Positives = 22/46 (48%), Gaps = 1/46 (2%)

Query  18   LNPRRDISSWLAQWFPRTPARSVVALKTPIKVELVAGKTYRWCVCG  63
            LNP     S L +W  + P  SV A K   ++E + GK   W  CG
Sbjct  352  LNPNHTPKSTLLKWSTKHPVPSVAAFKASYELEKIQGKRGIW-FCG  396


  Database: All non-redundant GenBank CDS translations+PDB+SwissProt+PIR+PRF
excluding environmental samples from WGS projects
    Posted date:  Nov 1, 2016  12:14 PM
  Number of letters in database: 1,663,633,117
  Number of sequences in database:  4,202,164

Lambda      K        H
   0.323    0.135    0.442
Gapped
Lambda      K        H
   0.267   0.0410    0.140
Matrix: BLOSUM62
Gap Penalties: Existence: 11, Extension: 1
Number of Sequences: 4202164
Number of Hits to DB: 13515519
Number of extensions: 491512
Number of successful extensions: 986
Number of sequences better than 100: 19
Number of HSP's better than 100 without gapping: 0
Number of HSP's gapped: 985
Number of HSP's successfully gapped: 19
Length of query: 127
Length of database: 1663633117
Length adjustment: 94
Effective length of query: 33
Effective length of database: 1268629701
Effective search space: 41864780133
Effective search space used: 41864780133
```

CISD3 with Higher plants BLAST

T: 11
A: 40
X1: 16 (7.5 bits)
X2: 38 (14.6 bits)
X3: 64 (24.7 bits)
S1: 40 (20.0 bits)
S2: 63 (28.9 bits)
ka-blk-alpha gapped: 1.9
ka-blk-alpha ungapped: 0.7916
ka-blk-alpha_v gapped: 42.6028
ka-blk-alpha_v ungapped: 4.96466
ka-blk-sigma gapped: 43.6362

BLASTP 2.5.1+

Reference: Stephen F. Altschul, Thomas L. Madden, Alejandro A. Schaffer, Jinghui Zhang, Zheng Zhang, Webb Miller, and David J. Lipman (1997), "Gapped BLAST and PSI-BLAST: a new generation of protein database search programs", Nucleic Acids Res. 25:3389-3402.

Reference for compositional score matrix adjustment: Stephen F. Altschul, John C. Wootton, E. Michael Gertz, Richa Agarwala, Aleksandr Morgulis, Alejandro A. Schaffer, and Yi-Kuo Yu (2005) "Protein database searches using compositionally adjusted substitution matrices", FEBS J. 272:5101-5109.

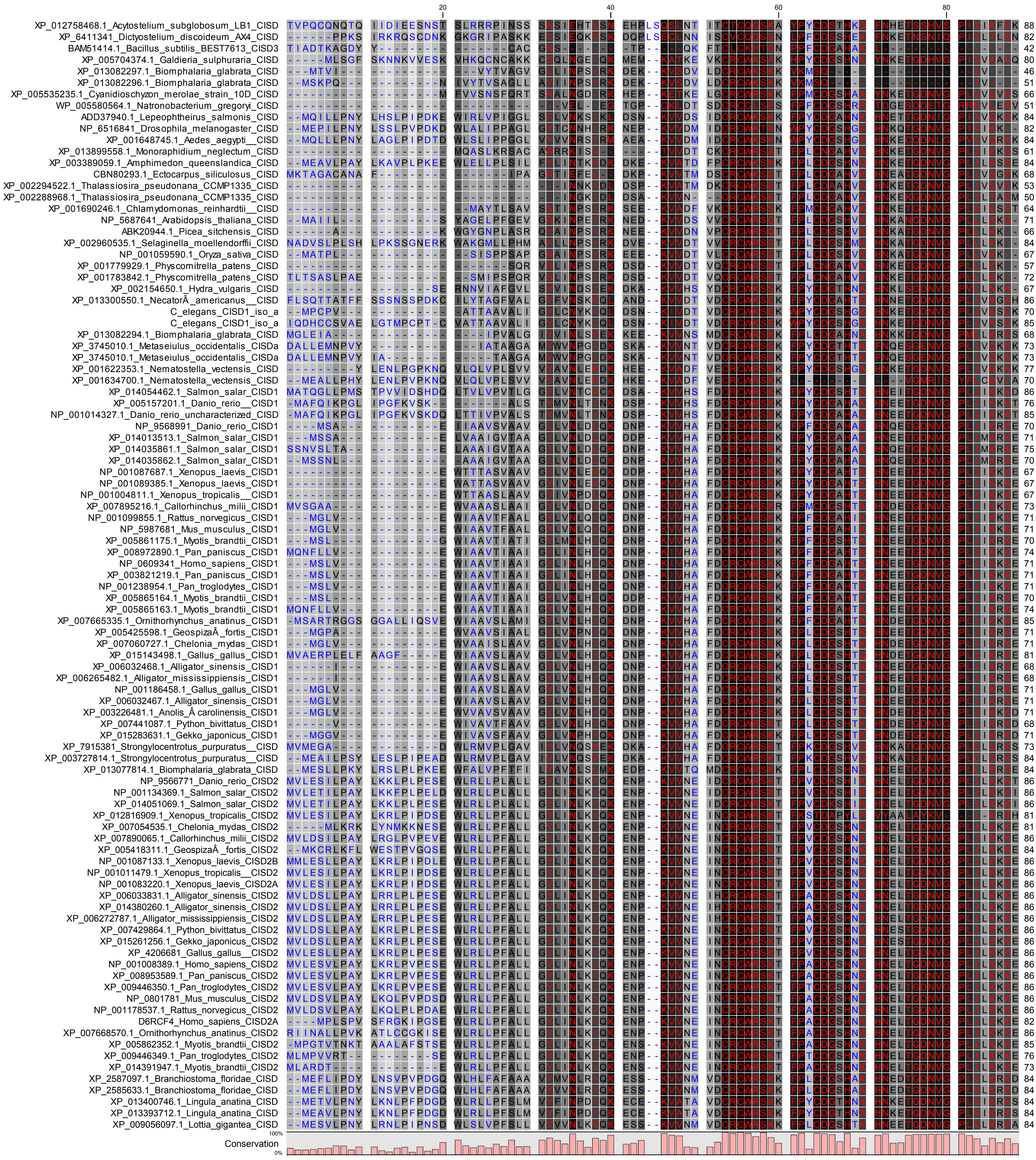

Supplementary Fig. S3. Multiple sequence alignment of CISD1 and CISD2 homologs generated using MUSCLE with default parameters. The bar graph represents the degree of conservation along the CDGSH domain. Color legend: *Background*: White – Least conserved, Black – Most conserved; *Font*: Blue – Least conserved; Red – Most conserved.

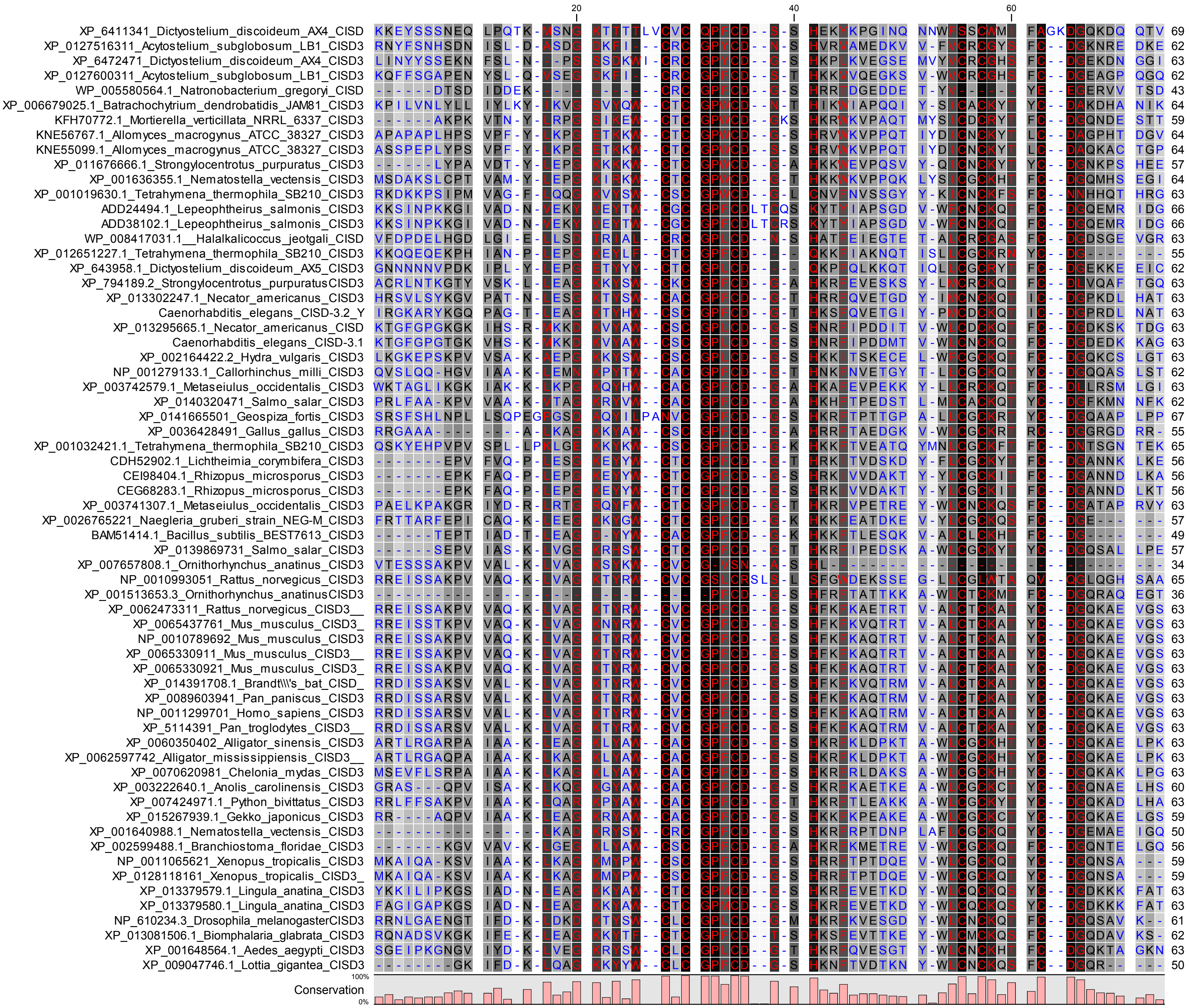

Supplementary Fig. S4. Multiple sequence alignment of CISD3 homologs generated using MUSCLE with default parameters. The bar graph represents the degree of conservation along the CDGSH domain. Color legend: *Background*: White – Least conserved, Black – Most conserved; *Font*: Blue – Least conserved; Red – Most conserved.

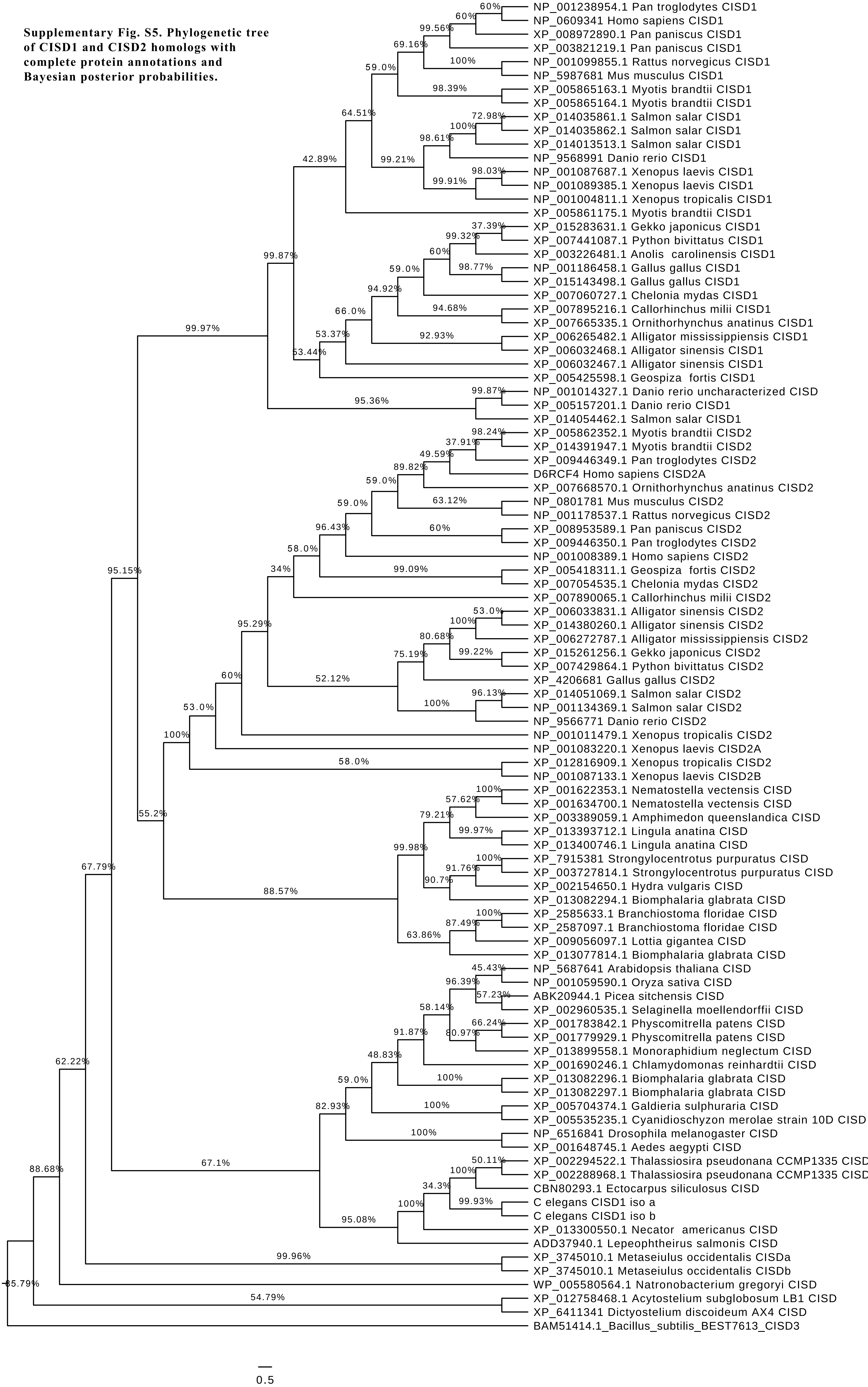

Supplementary Fig. S5. Phylogenetic tree of CISD1 and CISD2 homologs with complete protein annotations and Bayesian posterior probabilities.

Supplementary Fig. S6. Phylogenetic tree of CISD3 homologs with complete protein annotations and Bayesian posterior probabilities.

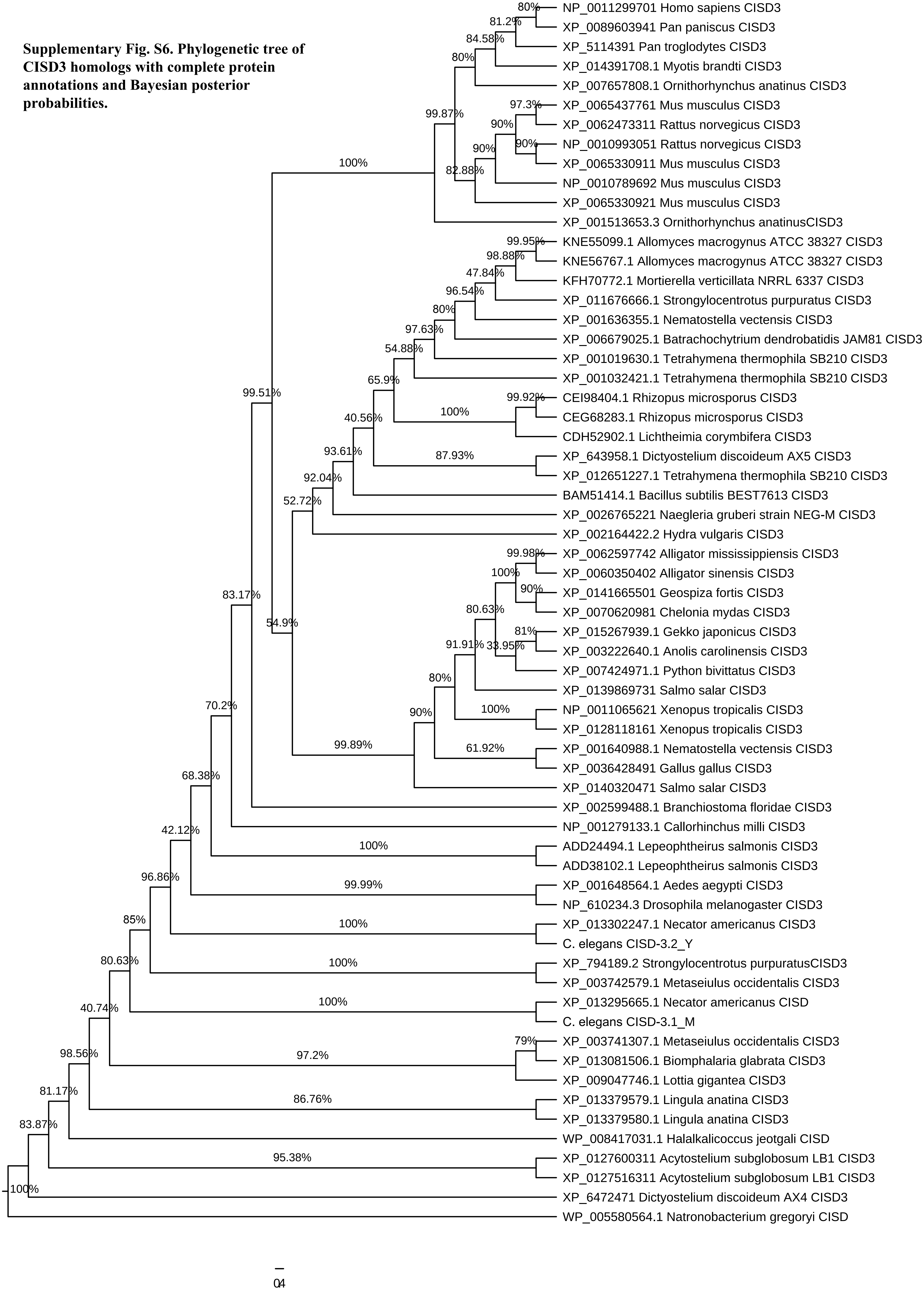

PSI- BLAST threshold	CISD 1 as Query			CISD 2 as Query			CISD 3 as Query		
	CISD 1	CISD 2	CISD 3	CISD 1	CISD 2	CISD 3	CISD 1	CISD 2	CISD 3
0.005	1	1	0	1	1	0	0	0	1
0.05	1	1	1	1	1	0	1	0	1
0.25	1	1	1	1	1	0	1	0	1
0.5	1	1	1	1	1	0	1	0	1
1	1	1	1	1	1	0	1	0	1
2	1	1	1	1	1	0	1	1	1
5	1	1	1	1	1	1	1	1	1
10	1	1	1	1	1	1	1	1	1

Supplementary Table S1. Sensitivity analysis used to set the optimum PSI-BLAST parameters. The homology between human CISD1, CISD2 and CISD3 proteins was determined (1 donates a hit, 0 donates a no hit) using a PSI-BLAST between the different proteins. Expect threshold value was set to 10 and PSI-BLAST threshold was altered until a BLAST with all three proteins identified a hits (1) with all three different proteins (i.e., all BLASTs resulted in a hit). This analysis set the lowest parameters that would obtain a hit with CISD1 or CISD2 on the more remotely homologous protein CISD3.

Supplementary Table S2 - Scaffold and contig N50 values for selected species.			● Represents completely sequenced genomes			
**Organism**	**Scaffold N50**	**Contig N50**	**Assembly**		**Assembly level**	**RefSeq category**
Homo sapiens	59364414	56413054	GCA_000001405.21		Chromosome	-
Pan troglodytes	8925874	50656	GCA_000001515.4		Chromosome	-
Pan paniscus	8197324	8197324	GCA_000258655.2		Chromosome	-
Rattus norvegicus	14986627	100461	GCA_000001635.6		Chromosome	-
Mus musculus	52589046	32273079	GCA_000001635.6		Chromosome	-
Myotis brandtii- Bat	3225832	23289	GCA_000412655.1		Scaffold	representative genome
Ornithorhynchus anatinus- Platypus	958970	11554	GCF_000002275.2		Chromosome	representative genome
Geospiza fortis	5255844	30521	GCA_000277835.1		Scaffold	representative genome
Gallus gallus - Bird	6379610	2894815	GCA_000002315.3		Chromosome	representative genome
Alligator sinensis - Reptile	2188296	23412	GCA_000455745.1		Scaffold	representative genome
Alligator mississippiensis Reptile		16610	GCA_000281125.3		Scaffold	representative genome
Chelonia mydas- Reptile	3864108	29240	GCA_000344595.1		Scaffold	representative genome
Python bivittatus -snakes- Reptile	213970	10658	GCA_000186305.2		Scaffold	representative genome
Anolis carolinensis-Lizard- Reptile	4033265	79867	GCA_000090745.2		Chromosome	representative genome
Gekko japonicus-Lizard- Reptile	707733	29574	GCA_001447785.1		Scaffold	representative genome
Xenopus (Silurana) tropicalis - Amphibian	124127367	72201	GCA_000004195.2		Scaffold	-
Xenopus laevis- Amphibian			Link		-	representative genome
Salmo salar - Fish		57618	GCA_000233375.4		chromosome	representive genome
Danio rerio-Fish	2181225	1258148	GCA_000002035.3		chromosome	reference genome
Callorhinchus milii - Cartilaginous fishes	4521921	46577	GCA_000165045.2		scaffold	representive genome
Branchiostoma floridae-Lancelet	2,586,727	27,925	GCA_000003815.1		scaffold	representive genome
Strongylocentrotus purpuratus - Echinoderm	419550	16785	GCA_000002235.3		scaffold	representive genome
Drosophila melanogaster		21485538	GCA_000001215.4		chromosome	reference genome
Aedes aegypti - Insect	1547048	82618	GCA_000004015.2		scaffold	representive genome
Lepeophtheirus salmonis - Crustaceans		16673	GCA_001005205.1		Contig	representive genome
Necator americanus - Worms	211861	5429	GCA_000507365.1		scaffold	representive genome
Caenorhabditis elegans - Worms		17493829	GCA_000002985.3		Complete Genome	reference genome
Lottia gigantea- Mollusca	1870055	96027	GCA_000327385.1		scaffold	representive genome
Biomphalaria glabrata - Mollusca	48059	7298	GCA_000457365.1		scaffold	representive genome
Lingula anatina-Brachipoda		53371	GCA_001039355.1		scaffold	representive genome
Nematostella vectensis - Cnidaria	472588	19835	GCA_000209225.1		scaffold	representive genome
Hydra vulgaris - Cnidaria	20916	10112	GCA_000004095.1		scaffold	representive genome
Amphimedon queenslandica - Sponge	120365	11817	GCA_000090795.1		scaffold	representive genome
Rhizopus microsporus-Fungi		30382	GCA_900000135.1		scaffold	representive genome
Lichtheimia corymbifera-Fungi		80219	GCA_000723665.1		scaffold	representive genome
Mortierella verticillata-Fungi	2912254	100238	GCA_000739165.1		scaffold	representive genome
Penicillium chrysogenum Fungi		4899068	GCA_000710275.1		chromosome	representive genome
Saccharomyces cerevisiae- Fungi			GCA_000146045.2	●	Complete Genome	reference genome
Batrachochytrium dendrobatidis (strain JAM81) (Frog chytrid fungus)	1484462	318114	GCA_000203795.1		scaffold	representive genome
Allomyces macrogynus- Fungi	1114524	35497	GCA_000151295.1		scaffold	representive genome
Oryza sativa Japonica – Plants Monocot		7131322	GCA_000005425.2		chromosome	-
Arabidopsis thaliana Plants Dicot		11194537	GCA_000001735.1		chromosome	reference genome
Picea sitchensis - Non - flowering plants - Pine			NC_011152.3		chloroplast	-
Selaginella moellendorffii - Fern	1749879	119796	GCA_000143415.2		scaffold	representive genome
Physcomitrella patens - Moss	1331933	74043	GCA_000002425.1		scaffold	representive genome
Monoraphidium neglectum - Sea weed algae	15659	9150	GCA_000611645.1		scaffold	representive genome
Chlamydomonas reinhardtii- Green algae	1695175	44607	GCA_000002595.2		scaffold	representive genome
Acytostelium subglobosum LB1 - Slime mould	889228	188563	GCA_000787575.2		scaffold	representive genome
Dictyostelium discoideum-Slime mould	5450249	341816	GCA_000004695.1		chromosome	representive genome
Acanthamoeba castellanii – Amoeba (Protozoan)	344205	44970	GCA_000313135.1		scaffold	representive genome
Galdieria sulphuraria - - Red algae	172322	116786	GCA_000341285.1		scaffold	representive genome
Cyanidioschyzon merolae strain 10D - Red algae			GCA_000091205.1	●	complete Genome	representive genome
Tetrahymena thermophila SB210 -Ciliophora - Rhizaria	486552	25644	GCA_000189635.1		scaffold	representive genome
Paramecium caudatum- Ciliophora		50166	GCA_000715435.1		scaffold	representive genome
Thalassiosira pseudonana CCMP1335 - Diatom		50166	GCA_000149405.2		chromosome	representive genome
Ectocarpus siliculosus - Brown algae	497380	32613	GCA_000310025.1		chromosome	representive genome
Naegleria gruberi – Amoeba (Protozoan)	401612	159679	GCA_000004985.1		scaffold	representive genome
Escherichia coli – Bacteria			GCA_000005845.2	●	complete Genome	reference genome
Pseudomonas fluorescens –Bacteria			GCA_000237065.1	●	complete Genome	representive genome
Bacillus subtilis - Bacteria			GCA_000009045.1	●	complete Genome	reference genome
Natronobacterium gregory – Archaea sps			GCA_000230715.3	●	complete Genome	representive genome
Halalkalicoccus jeotgali – Archaea sps			GCA_000196895.1	●	complete Genome	representive genome

Supplementary Table S3 -CISD1 and CISD2 homologs and their GeneID. Highlighted rows indicate protein variants encoded by same gene (alternative splicing)

Accession no.	Organism name	GeneID	Locus Tag	Chromosome	Start position	End position	Exon count	
XP_012758468.1	Acytostelium subglobosum LB1	24516374			690505	691623	1	
XP_001648745.1	Aedes aegypti	5564392			1963516	1978128	4	
XP_006265482.1	Alligator mississippiensis	102559029		Unknown	320591	339137	3	
XP_006272787.1	Alligator mississippiensis	102574509		Unknown	5809127	5825243	3	
XP_006032467.1	Alligator sinensis	102377599		Unknown	644392	666208	4	
XP_006032468.1	Alligator sinensis	102377599		Unknown	644392	666208	4	
XP_006033831.1	Alligator sinensis	102369072		Unknown	644392	666208	4	
XP_014380260.1	Alligator sinensis	102369072		Unknown	644392	666208	4	
XP_003389059.1	Amphimedon queenslandica	100631667		Unknown	46425	47325	4	
XP_003226481.1	Anolis carolinensis	100559354		Unknown	115758	123903	3	
NP_5687641	Arabidopsis thaliana	835246		5	21009592	21010345	2	
XP_013082294.1	Biomphalaria glabrata	106063883		Unknown	204180	211794	4	
XP_013077814.1	Biomphalaria glabrata	106067631		Unknown	39871	43349	3	
XP_013082296.1	Biomphalaria glabrata	106067632		Unknown	47632	50119	3	
XP_013082297.1	Biomphalaria glabrata	106067632		Unknown	47632	50119		
XP_2587097.1	Branchiostoma floridae	7209528			336660	0.340664	5	
XP_2585633.1	Branchiostoma floridae	7238551			57124	61212	5	
CAI46628.1	Caenorhabditis elegans CISD1 isoform a	3565570		2	11468552	11469624	3	
CAO82066.1	Caenorhabditis elegans CISD1 isoform b	3565570		2	11468552	11469624	2	
XP_007895216.1	Callorhinchus milii	103177628		Unknown	832427	843209	3	
XP_007890065.1	Callorhinchus milii	103180914		Unknown	5450839	5455628	3	
XP_007054535.1	Chelonia mydas	102933650		Unknown	729124	741699	3	
XP_007060727.1	Chelonia mydas	102943977		Unknown	258424	302718	3	
XP_001690246.1	Chlamydomonas reinhardtii	5715740			5067028..	5068245	4	
XP_005535235.1	Cyanidioschyzon merolae strain 10D	16992357		2	425376	425699	1	
NP_9568991	Danio rerio	393354		1	42985123	42990977	3	
NP_9566771	Danio rerio	393577		13	41784372	41791059	3	
XP_005157201.1	Danio rerio	541492		14	7596241	7610720	3	
NP_001014327.1	Danio rerio	541492		14	7596241	7610720	3	
XP_6411341	Dictyostelium discoideum AX4	8622692		3	3657513	3658445	1	
NP_6516841	Drosophila melanogaster	43459		3R	29222217	29223428	3	
CBN80293.1	Ectocarpus siliculosus		Esi_0052_0037					
XP_005704374.1	Galdieria sulphuraria	17086734		Unknown	109761	110391	3	
XP_4206681	Gallus gallus	422714		4	61334093	61340503	3	
NP_001186458.1	Gallus gallus	423642		6	5238502	5248084	4	
XP_015143498.1	Gallus gallus	423642		6	5238502	5248084	4	
XP_015283631.1	Gekko japonicus	107105755		Unknown	676405	696365	3	
XP_015261256.1	Gekko japonicus	107124654		Unknown	93575	99554	3	
XP_005418311.1	Geospiza fortis	102032512		Unknown	694777	707714	4	
XP_005425598.1	Geospiza fortis	102036457		Unknown	15422496	15429582	3	
NP_001008389.1	Homo sapiens	55847		10	58269102	58289259	3	
NP_0609341	Homo sapiens	493856		4	102868978	102892807	3	
D6RCF4	Homo sapiens							
XP_002154650.1	Hydra vulgaris	100208174		Unknown	108866	120214	3	
ADD37940.1	Lepeophtheirus salmonis				55	498		
XP_013400746.1	Lingula anatina	106161326		Unknown	447985	450542	3	
XP_013393712.1	Lingula anatina	106166640		Unknown	16401	19118	3	
XP_009056097.1	Lottia gigantea	20246628		Unknown	2816000	2819823	3	
XP_3745010.1	Metaseiulus occidentalis							
XP_3745010.1	Metaseiulus occidentalis	100904812		Unknown	250172	255975	4	
XP_013899558.1	Monoraphidium neglectum	25740300		Unknown	7966	9041	4	
NP_5987681	Mus musculus	52637		10	71330494	71344849	3	
NP_0801781	Mus musculus	67006		3	135406412	135424442	4	
XP_005865164.1	Myotis brandtii	102242547		Unknown	1939147	1959988	5	
XP_005865163.1	Myotis brandtii	102242547		Unknown	1939147	1959988	5	
XP_005861175.1	Myotis brandtii	102252862		Unknown	568959	585919	5	
XP_014391947.1	Myotis brandtii	102252862		Unknown	568959	585919	5	
XP_005862352.1	Myotis brandtii	102255750		Unknown	5518314	5518767	1	
WP_005580564.1	Natronobacterium gregoryi				1808627	1808866		
XP_013300550.1	Necator americanus	25350452		Unknown				
XP_001622353.1	Nematostella vectensis	5500979					2	Hypothetical Protein
XP_001634700.1	Nematostella vectensis	5514529					1	Hypothetical Protein
XP_007665335.1	Ornithorhynchus anatinus	100075406		7	36420461	36434705	5	
XP_007668570.1	Ornithorhynchus anatinus	100076364		Unknown	1873990	1891686	3	
NP_001059590.1	Oryza sativa							
XP_008972890.1	Pan paniscus	100973439		10	57020351	57041928	4	
XP_003821219.1	Pan paniscus	100973439		10	57020351	57041928	4	
XP_008953589.1	Pan paniscus	103783527		4	105954880	105975142	3	
XP_009446349.1	Pan troglodytes	748227		10	56608271	56626778	3	
XP_009446350.1	Pan troglodytes	100609495		4	105374400	105437958	4	
NP_001238954.1	Pan troglodytes	100609495		4	105374400	105437958	4	
XP_001779929.1	Physcomitrella patens	5943135			413841	414477	2	Hypothetical protein
XP_001783842.1	Physcomitrella patens	5947048			93153	93783	2	
ABK20944.1	Picea sitchensis							
XP_007441087.1	Python bivittatus	103050463		Unknown	185590	199912	3	

XP_007429864.1	Python bivittatus	103050464		Unknown	185590	199912	3		
NP_001099855.1	Rattus norvegicus	294362		20	18493538	18506919	3		
NP_001178537.1	Rattus norvegicus	295457		2	240586754	240611560	3		
NP_001134369.1	Salmo salar	100195868		ssa08	5947157	5952849	3		
XP_014013513.1	Salmo salar	106578835		ssa19	38655706	38660624	3		
XP_014035861.1	Salmo salar	106589936		ssa28	34965366	34971099	3		
XP_014035862.1	Salmo salar	106589936		ssa28	34965366	34971099	3		
XP_014051069.1	Salmo salar	106602754		ssa04	22362212	22366780	3		
XP_014054462.1	Salmo salar	106604402		ssa05	15340587	15349207	3		
XP_002960535.1	Selaginella moellendorffii	9642277		Unknown					
XP_7915381	Strongylocentrotus purpuratus	586672		Unknown	5143433	5145853	9		
XP_003727814.1	Strongylocentrotus purpuratus	586672		Unknown	5143433	5145853	9		
XP_002294522.1	Thalassiosira pseudonana CCMP1335	7449541		3	1707375	1707569	1		
XP_002288968.1	Thalassiosira pseudonana CCMP1335	7453077		17	517638	517835	1		
NP_001087687.1	Xenopus laevis	398808							
NP_001089385.1	Xenopus laevis	447022							
NP_001083220.1	Xenopus laevis	447511							
NP_001087133.1	Xenopus laevis	734435							
NP_001004811.1	Xenopus tropicalis	496970		Unknown	149811492	149820273	4		
NP_001011479.1	Xenopus tropicalis	496970		Unknown			4		
XP_012816909.1	Xenopus tropicalis	448057		Unknown					

Supplementary Table S3 -CISD3 homologs and their GeneID. Highlighted rows indicate protein variants encoded by same gene (alternative splicing)

Accession number	Organism name	GeneID	Locus Tag	Chromosome	Start position	End position	Exon count	
XP_012751631.1	Acytostelium subglobosum LB1	24519012			869137	869646	2	
XP_012760031.1	Acytostelium subglobosum LB1	24524146			600351	600869	2	
XP_001648564.1	Aedes aegypti	5564228		2	250129	250894	4	
XP_006259774.2	Alligator mississippiensis	102574652		Unknown	1408888	1418284	3	
XP_006035040.2	Alligator sinensis	102373679		Unknown	53341	62669	3	
KNE56767.1	Allomyces macrogynus ATCC 38327		AMAG_02543					hypothetical protein
KNE55099.1	Allomyces macrogynus ATCC 38327		AMAG_01033					hypothetical protein
XP_003222640.1	Anolis carolinensis	100556592		6	72972201	72978983	3	
BAM51414.1	Bacillus subtilis BEST7613	14354624			2598432	2598668		
XP_006679025.1	Batrachochytrium dendrobatidis JAM81	18238696		Unknown	1058282	1059011	4	
XP_013081506.1	Biomphalaria glabrata	106066951		Unknown	19386	25009	3	
XP_002599488.1	Branchiostoma floridae	7227660			1082967	1096804	2	
NP_001279133.1	Callorhinchus milii	103185693		Unknown	27625	32587	2	
XP_007062098.1	Chelonia mydas	102944443		Unknown	7410561	7420692	3	
XP_6472471	Dictyostelium discoideum AX4	8616052		1	675419	675964	2	
XP_643958.1	Dictyostelium discoideum AX4	8619386		2	3981595	3982101	1	
NP_610234.3	Drosophila melanogaster	35585		2R	6756296	6757209	4	
NP_497920.3	Caenorhabditis elegans CISD-3.1	175593		3	4542882	4545782	4	
NP_497419.1	Caenorhabditis elegans CISD-3.2	175307		3	1540506	1543304	4	
XP_003642849.1	Gallus gallus	100857847		27	4421720	4422397	2	
XP_015267939.1	Gekko japonicus	107111489		Unknown	1572245	1578763	3	
XP_014166550.1	Geospiza fortis	102031810		Unknown	51036	83006	20	
WP_008417031.1	Halalkalicoccus jeotgali	9419879		Unknown	1922282	1922971		
NP_001129970.1	Homo sapiens	284106		17	38730257	38735605	4	
XP_002164422.2	Hydra vulgaris	100197388		Unknown				
ADD24494.1	Lepeophtheirus salmonis	BT121172.1			82	540		hypothetical protein
ADD38102.1	Lepeophtheirus salmonis	BT120854			82	540		hypothetical protein
CDH52902.1	Lichtheimia corymbifera		LCOR_04333.1					
XP_013379579.1	Lingula anatina	106151048		Unknown	135795	138541	4	
XP_013379580.1	Lingula anatina	106151048		Unknown	135795	138541	4	
XP_009047746.1	Lottia gigantea	20253312		Unknown	381455	382004	2	
XP_003741307.1	Metaseiulus occidentalis	100905571		Unknown	30642	31415	2	
XP_003742579.1	Metaseiulus occidentalis	100909263		Unknown	38604	39581	2	
KFH70772.1	Mortierella verticillata NRRL 6337		MVEG_03620					hypothetical protein
XP_006543776.1	Mus musculus	217149		11	97685504	97688629	4	
NP_001078969.2	Mus musculus	217149		11	97685504	97688629	4	
XP_006533091.1	Mus musculus	217149		11	97685504	97688629	4	
XP_006533092.1	Mus musculus	100504524		2	162919088	162923272	1	
XP_014391708.1	Myotis brandtii	102251477		Unknown	5591110	5635216	23	
XP_002676522.1	Naegleria gruberi strain NEG-M	8856590					3	hypothetical protein
XP_013302247.1	Necator americanus	25342537		Unknown	583536	587496	8	
XP_013295665.1	Necator americanus	25353555		Unknown	51717	53812	5	
XP_001636355.1	Nematostella vectensis	5516262					3	hypothetical protein
XP_007657808.1	Ornithorhynchus anatinus	103166705		Unknown	199	406	1	
XP_008960394.1	Pan paniscus	100973823		17	18815044	18820685	4	
XP_5114391	Pan troglodytes	454613		17	18660924	18664278	4	
XP_007424971.1	Python bivittatus	103048605		Unknown	239683	242946	3	
NP_001099305.1	Rattus norvegicus	287661		10	85628596	85631656	4	
XP_006247331.1	Rattus norvegicus	287661		10	85628596	85631656	4	
CEI98404.1	Rhizopus microsporus		RMCBS344292_12513					hypothetical protein
CEG68283.1	Rhizopus microsporus		RMATCC62417_04577					hypothetical protein
XP_014032047.1	Salmo salar	106564950		ssa12	23066233	23068342	3	
XP_013986973.1	Salmo salar	106587910		ssa02	46100586	46102925	3	
XP_794189.2	Strongylocentrotus purpuratus	589458		Unknown	308726	312642	3	
XP_011676666.1	Strongylocentrotus purpuratus	100889608		Unknown	171788	181890	4	
XP_001032421.1	Tetrahymena thermophila SB210	7832838			264844	265570	3	
XP_001019630.1	Tetrahymena thermophila SB210	7829264			908503	909508	2	
XP_012651227.1	Tetrahymena thermophila SB210	24437639			42718	43487	5	
XP_012811816.1	Xenopus tropicalis	100127760		Unknown	295527	301099	4	
NP_001106562.1	Xenopus tropicalis	100127760		Unknown	295527	301099	4	
